# Picture quiz

**Published:** 2019-05-13

**Figure:**
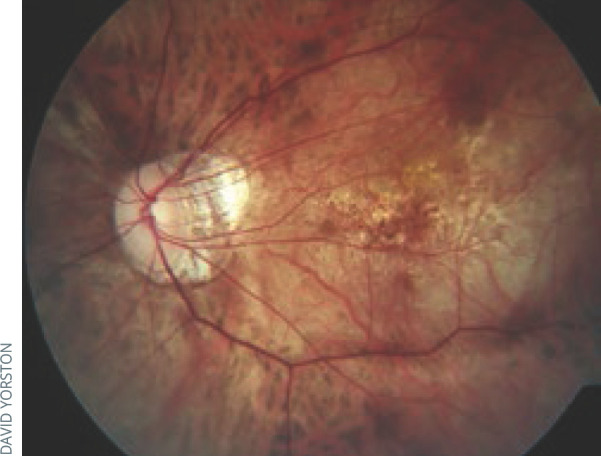

**Tick ALL that are TRUE**

**Question 1 What is wrong with this eye?**
  □ **a.** Dry age-related macular degeneration
  □ **b.** Retinitis pigmentosa
  □ **c.** High myopia
  □ **d.** Primary open-angle glaucoma
**Question 2 Which of the following are more common in patients with this condition?**
  □ **a.** Retinal detachment
  □ **b.** Choroidal neovascularisation
  □ **c.** Acute angle-closure glaucoma
  □ **d.** Open-angle glaucoma
  □ **e.** Ptosis
**Question 3 Which of the following have been shown to delay the onset or the progression of this condition?**
  □ **a.** Not wearing spectacles
  □ **b.** Avoiding playing sports
  □ **c.** Using low-dose atropine drops
  □ **d.** Spend more time outside
  □ **e.** Avoiding sunlight

## ANSWERS

1. c. It is high myopia. There is tilting of the optic disc with chorio-retinal atrophy around the optic disc and throughout the fundus, associated with a pale retina due to atrophy of the retinal pigment epithelium.
2. a, b and d. Retinal detachment, choroidal neovascularisation and open angle glaucoma are all associated with high myopia.
3. c and d. Spending more time outside has been shown to prevent or delay the onset of myopia and low-dose atropine has been shown to reduce progression of myopia in school-aged children.

